# Normative data and predictors of trunk muscle endurance performance in young tennis players

**DOI:** 10.3389/fspor.2026.1804617

**Published:** 2026-06-03

**Authors:** Dario Novak, Velibor Viboh, Ivan Belcic, Zlatan Bilic, Goran Markovic

**Affiliations:** 1 Faculty of Kinesiology, University of Zagreb, Zagreb, Croatia; 2 Institute of Sport Science and Innovations, Lithuanian Sports University, Kaunas, Lithuania; 3 Polyclinic Medical Body Balance, Zagreb, Croatia; 4 Faculty of Chemical Engineering and Technology, University of Zagreb, Zagreb, Croatia; 5 Motus Melior Ltd., Zagreb, Croatia

**Keywords:** athletic profiling, physical performance, sex differences, talent identification, youth tennis

## Abstract

**Introduction:**

Trunk muscle endurance is important for injury prevention and performance in tennis due to repetitive rotational and high-range motion loads. Normative data for youth tennis players is have not been established and the influence of body size and competitive rank on these measures remains unclear. The aim of this study was to establish age- and sex-specific reference values for trunk endurance and to examine the influence of body size and competitive success on endurance capacity

**Methods:**

A total of 214 young tennis players (115 males, 99 females; age=14.1 ± 2.3 years) registered with the Croatian Tennis Association. Participants were stratified by national ranking into high-achieving (HA; ranked top 30) and low-achieving (LA) groups. Trunk flexor endurance was measured using the plank test, and trunk extensor endurance was measured using the Biering-Sørensen (s). Covariates included age, sex, height, and weight.

**Results:**

In female players, HA participants demonstrated significantly better trunk extensor endurance than LA participants (F = 5.35, *p* = 0.023) after controlling for body size. No significant differences in endurance were found between HA and LA males (*p* > 0.05). Regression analysis showed that body height and weight were not significantly associated with test performance in either sex (*p* = 0.08–0.98). Females in the U18 category significantly outperformed males in the Biering-Sørensen test (*p* < 0.01). The key findings of the present study indicate that neither height nor weight are significantly correlated with the core strength and endurance of the back muscles in both male and female young tennis players. Player level significantly influences the results of the plank and Biering-Sørensen tests in females, whereas in males, differences are not statistically significant after accounting for body size.

**Discussion:**

These results highlight the importance of structured trunk endurance training, particularly for female players, where core performance appears to be closely related to competitive success. Age and sex-specific normative values for these tests may assist coaches and researchers in evaluating, monitoring, and developing core strength and endurance in youth tennis populations, with potential implications for improving performance and injury prevention.

## 1 Introduction

Adequate trunk muscle endurance is important for injury prevention and performance in sports (1, 10, 25). This is particularly relevant in tennis, which involves repetitive trunk extensions, lateral flexions, and rotations across large ranges of motion (6, 7). Low back pain and spinal injuries are common in tennis players (15, 24) and given their high recurrence and recovery demands (17), they can have lasting effects on an athlete's career. Thus, beyond load management, coaches should systematically assess and train trunk muscle endurance in young players. In tennis-specific movement patterns, trunk muscles play a central role in force transmission between the lower and upper extremities, contributing to stroke efficiency, postural control, and mechanical load distribution along the kinetic chain (21). Insufficient trunk endurance may therefore result not only in reduced performance efficiency but also in increased mechanical stress on the lumbar spine and surrounding structures during repeated high-intensity actions such as serves and groundstrokes.

Isometric endurance tests provide a simple and inexpensive method of evaluating trunk muscle function in both clinical and sport settings (12, 18). Although there are a variety of tests (12, 20, 27), they primarily target flexors and extensors, and normative data in young tennis players remain scarce. In fact, trunk endurance tests have not typically been included in standard fitness assessments for this population (13, 28, 29).

The lack of normative data is particularly problematic in youth tennis, where pronounced interindividual differences in biological maturation, growth velocity, and training exposure are commonly observed. During adolescence, rapid changes in body dimensions, segmental proportions, and neuromuscular coordination may transiently affect movement efficiency and load tolerance (19). Such developmental variability complicates the interpretation of physical performance tests, as observed differences may reflect maturational status rather than true training-induced adaptations.

From a talent development perspective, this issue is especially relevant, as physical test results are often used to support selection and training decisions in youth sport. Without appropriate normative benchmarks, there is a risk of misclassifying athletes by overestimating or underestimating their physical capacities, particularly during sensitive periods of growth and maturation. Establishing age- and sex-specific normative values may therefore support evidence-based training prescription, talent development, and early identification of players who may benefit from targeted trunk conditioning interventions.

To address this gap, we conducted a cross-sectional study in 214 male and female Croatian tennis players aged 11–17 years. We evaluated trunk flexor and extensor endurance, examined the influence of body size while controlling for age, and tested whether endurance capacity can distinguish more successful players from less successful players. Finally, we established age- and sex-specific reference values for use in training and talent development. In addition to providing descriptive reference data, this approach allows for evaluation of the discriminative value of trunk endurance measures in relation to competitive performance level.

We hypothesised that (a) males would outperform females in trunk endurance across all age categories (5); (b) body height and weight would be negatively associated with endurance when controlling for age; and (c) trunk endurance, independent of body size, would discriminate successful players from less successful.

## 2 Methods

### 2.1 Subjects

The study included 214 young tennis players (115 males, 99 females) aged 11–17 years (mean ± SD: 14.1 ± 2.3 years). All participants were registered members of the Croatian Tennis Association. Players were stratified according to their national ranking: high-performing players were defined as those ranked 30th or higher, while lower-performing players were ranked below 30th or unranked.

### 2.2 Sample size estimation and justification

The required sample size for this cross-sectional study (*N* = 214) was calculated *a priori* using G*Power statistical software (version 3.1.9.4). The determination was based on ensuring adequate statistical power (0.80), standard Type I error rate (0.05), effect size (0.25) and number of groups (8) and covariates (2). The calculated required sample size shows 179 participants for both ANOVA and ANCOVA and 92 for regression analysis. The sample of 214 young tennis players exceeded the required minimum sample for all primary analyses with the specified parameters, confirming that the study is sufficiently powered to detect a medium-sized effect if present, and it is highly powered in multiple regression analysis.

### 2.3 Procedure

The endurance of the trunk flexor was assessed using the plank test, in which participants maintain a prone position supported by the elbows and feet, keeping the body in a straight line from head to heels (14). Back extensor endurance was evaluated using the Biering-Sørensen test, a widely used measure of paraspinal and hip extensor endurance in both athletes and the general population (11). Participants were required to maintain a horizontal position of the head, arms, and trunk against gravity for as long as possible (4). Both tests were recorded in seconds, with a single trial performed for each. The characteristics of the participants were also recorded, including age, sex, height, weight, and level of play.

### 2.4 Statistical analysis

Descriptive statistics (mean values, SD, and percentiles) stratified by age and sex were calculated to establish normative values for core strength and endurance capacity of the back muscles in participants. Prior to inferential analyses, the assumptions of normality and homogeneity of variance were examined using the Shapiro–Wilk test and Levene's test, respectively. A two-way univariate ANOVA was used to analyse the main effects of sex and age group in each trunk endurance test, as well as the interaction effect between sex and age group. In case of a significant interaction effect, a *post-hoc* independent t-test was used to compare males vs. females within each age group. Multiple regression analyses were conducted to explore the association between height and weight, and performance in plank and Biering-Sørensen tests in both male and female young tennis players, with age being a covariate. The differences in plank and Biering-Sørensen tests results between high- and low-achievers were analysed using analysis of covariance (ANCOVA), with weight and height included as covariates. All statistical analyses were performed using IBM SPSS Statistics for Windows, Version 24.0 (IBM Corp., Armonk, NY, USA) with the threshold for statistical significance set to *p* < 0.05.

## 3 Results

Table 1 presents the characteristics of the participants. The overall study population had a mean age of 14.1 years, a mean height of 165.5 cm, and a mean weight of 55.2 kg. Right-hand dominance was observed in 196 participants, while 17 were left-hand dominant. The sample was divided into 100 high-achieving players and 114 low-achieving players.

**Table 1:** Participant characteristics.

Variable	Participants (*N* = 214)
Age, y	
All, mean (SD)	14.10 (2.32)
Male, mean (SD)	14.05 (2.32)
Female, mean (SD)	14.15 (2.32)
Sex	
Male, N (%)	115 (54)
Female, N (%)	99 (46)
Height, cm	
All, mean (SD)	165.54 (13.34)
Male, mean (SD)	168.04 (13.95)
Female, mean (SD)	162.56 (11.92)
Weight, kg	
All, mean (SD)	55.22 (12.88)
Male, mean (SD)	56.92 (14.18)
Female, mean (SD)	53.25 (10.92)
Playing level	
High achievers N (%)	100 (47)
Low achievers N (%)	114 (53)
Dominant hand	
Right, N (%)	196 (92)
Left, N (%)	17 (8)

Legend: N, number of participants; SD, standard deviation.

Mean (SD) plank and Biering-Sørensen tests performance for high-achieving players (HA) were 136.4 (60.5) s and 127.7 (48.8) s, while the corresponding results for low-achieving players (LA) were 120.1 (69.6) s and 110.5 (40.1) s, respectively.

Table 2 presents mean, standard deviation, and percentile values (representing normative values) for Plank and Biering-Sørensen tests, in seconds, stratified by age and sex.

**Table 2:** Mean values, *SD*s, and percentile values (s) for plank and biering- sørensen tests according to age and sex .

Sex	Test	Age category	N	Mean (s)	SD (s)	P5 (s)	P10 (s)	P25 (s)	P50 / Median (s)	P75 (s)	P90 (s)
M	Plank	U12	37	138.0	107.6	49.0	59.6	80.5	102.0	149.0	300.2
		U14	24	109.1	46.7	36.3	40.0	83.8	106.0	125.0	177.5
		U16	32	141.8	47.3	58.9	77.8	106.5	131.5	180.0	180.0
		U18	22	131.0	75.7	18.9	42.5	78.0	122.0	181.0	268.0
	Bie-Sor	U12	37	114.7	52.3	38.7	48.8	83.5	120.0	127.0	186.4
		U14	24	100.6	37.0	23.0	47.5	72.0	103.5	124.0	148.0
		U16	32	119.6	34.7	65.3	69.9	88.5	123.0	144.5	178.2
		U18	22	81.5	29.4	25.8	35.4	64.0	80.0	96.3	123.7
F	Plank	U12	34	110.5	70.8	32.00	46.50	62.25	99.50	130.75	218.00
		U14	18	114.3	40.0	55.0	59.5	86.0	118.0	145.2	178.4
		U16	24	115.9	34.8	64.8	71.0	82.5	120.0	125.0	176.0
		U18	22	121.9	53.8	59.2	60.9	88.5	101.5	142.5	229.3
	Bie-Sor	U12	34	130.9	43.6	47.0	79.5	106.8	131.5	158.0	183.5
		U14	19	126.1	50.9	70.0	70.0	85.0	113.0	180.0	184.0
		U16	24	113.8	31.4	67.5	75.0	85.5	116.5	127.5	168.0
		U18	22	122.5	39.5	63.5	68.1	96.5	120.0	138.0	181.0

Legend: N, number of participants; SD, standard deviation; P5, 5th percentile; P10, 10th percentile; P25, 25th percentile; P50, 50th percentile/median; P75, 75th percentile; P90, 90th percentile.

**Figure 1:**
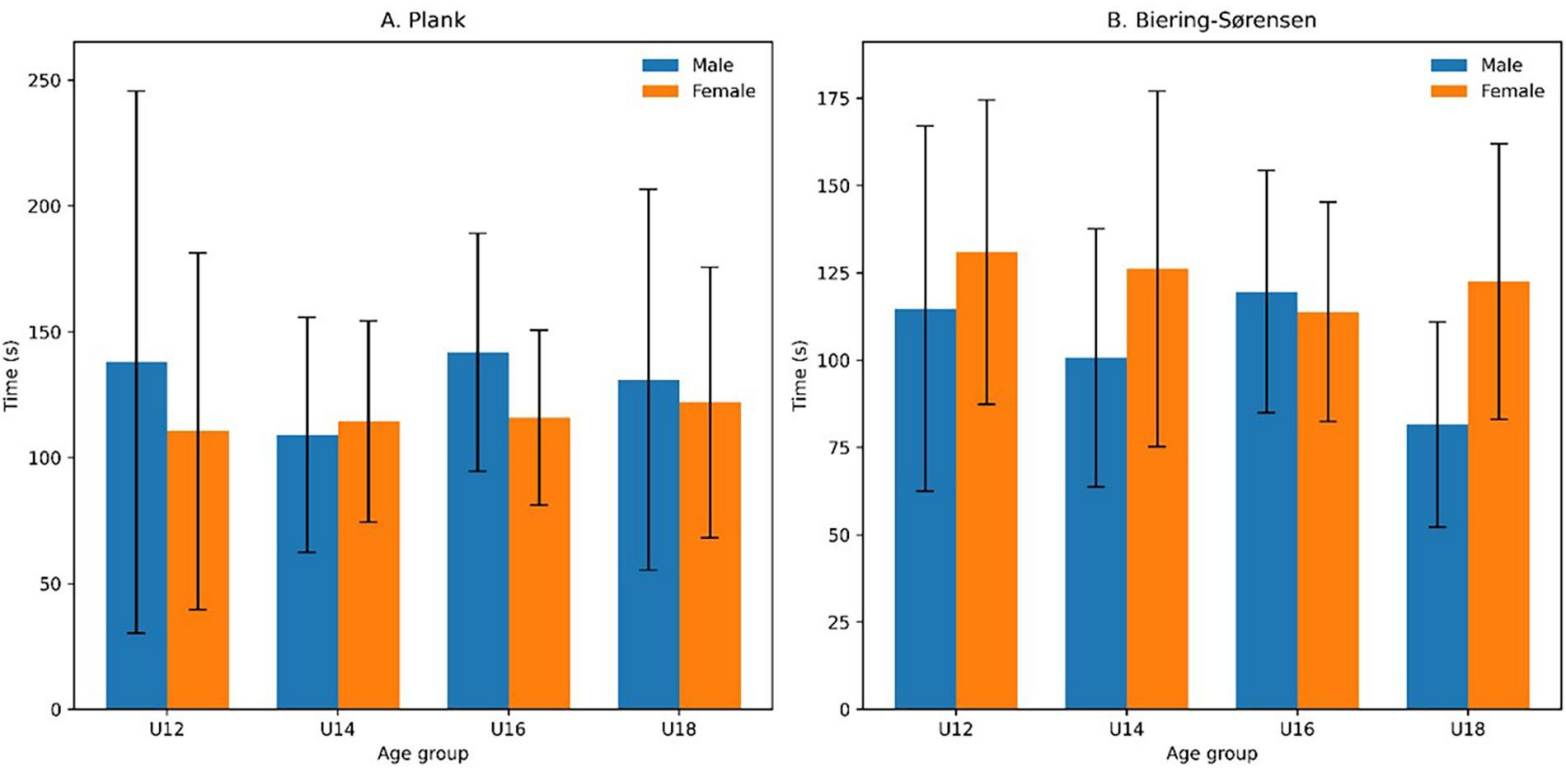
Mean (SD) results in plank **(A)** and biering-sorensen tests **(B)** for male and female tennis players according to age. *Significant differences within age group (*p* < 0.01).

Two-way ANOVA analyses revealed significant sex x age interaction effect for Biering- Sørensen test (*p* = 0.042), but not for plank test (*p* = 0.059). Significant main effect of sex was found for Biering- Sørensen test (*p* = 0.001), while all other main effects were not significant (*p* = 0.07–0.64). *post-hoc* comparison test revealed significantly higher (*p* < 0.01) performance in Biering- Sørensen test in female vs. male players in U18 age group. Figure 1 shows the performance in both trunk endurance tests for both sexes according to age group.

The results of regression analyses with plank and Biering- Sørensen test as criterion variables revealed that in both males and females, body height and weight were not significantly associated with plank and Biering- Sørensen test (males: plank R = 0.12, Biering-Sørensen R = 0.16; females: plank R = 0.09, Biering-Sørensen R = 0.14; all *p* = 0.08–0.98). After controlling for body height and weight, the ANCOVA analyses revealed no significant differences between high- and low-achievers in males in either plank or Biering-Sørensen performance (Plank: F = 2.96, *p* = 0.088; Biering-Sørensen: F = 0.37, *p* = 0.544). In females, no significant difference was found for plank endurance (F = 0.76, *p* = 0.387), while a significant difference was observed for the Biering-Sørensen test (F = 5.35, *p* = 0.023), with high-achievers demonstrating better trunk extensor endurance.

## 4 Discussion

The first hypothesis was not supported, as trunk muscle endurance did not show a consistent age-related increase across all subgroups. However, when analysing female participants specifically, the results indicate a continuous improvement in the plank test with age. In contrast, the Biering-Sørensen endurance test results for U16 girls did not align with this expected trend, whereas the U12, U14, and U18 age groups consistently demonstrated better performance on this test.

When examining sex-based differences, it was observed that male players generally outperformed female players in the categories U12, U16, and U18, except for the U14 category, where the trend was reversed. A possible explanation for this result could be the earlier onset of puberty in girls, which could contribute to an accelerated development of strength during this phase of growth (16). During and shortly after the period of fastest growth, adolescents often experience temporary alterations in coordination, movement efficiency, and muscular control due to rapid changes in body dimensions and segment proportions. In female players, who generally enter and complete pubertal development earlier than males, interindividual differences in maturity timing within the same chronological age category may be particularly pronounced. Similar conclusion and results were obtained by Söğüt et al. (26), where authors suggest that physical size and advanced maturity should be taken into consideration, especially at girls’ U14 level and in the selection and identification of youth elite female tennis players. The previous study does not align with this research, as the results show no statistically significant difference between sexes in the Biering-Sørensen strength test in a sample of physically active students (30), as well as in emergency task force groups, athlete groups, university students, and non-elite police and firefighters (22).

The results obtained completely refute the second hypothesis. When analysing the influence of body size, specifically height and weight on the performance in the plank and Biering-Sørensen tests, (while considering each sex separately and adjusting for age) no significant associations were observed. This suggests that anthropometric characteristics, at least within the normal developmental range of 11–17 years, do not play a decisive role in determining trunk muscle endurance among young tennis players. These findings are consistent with previous reports indicating that isometric endurance tests are less dependent on body size than on muscle activation strategies and fatigue resistance (20, 27). Technical proficiency in test execution, neuromuscular efficiency, and motor control may outweigh the influence of absolute body mass or stature. Furthermore, the absence of association with body size implies that both taller and shorter athletes, as well as lighter and heavier players, can develop comparable levels of trunk endurance, provided similar levels of training exposure. This may be particularly relevant in youth tennis, where large interindividual variations in growth and maturation exist, yet endurance capacity of the trunk appears relatively robust against such differences. In contrast, in sports such as gymnastics or rowing, where body size and limb length strongly influence leverage and force application, anthropometric factors often play a much greater role in physical performance (2, 8). The lack of such associations in tennis suggests that trunk endurance represents a quality that can be developed equally between different body types. Therefore, factors such as training history, muscle fibre composition, or even genetic predispositions may be more important determinants of performance in these tests than body size *per se*. From a practical point of view, this indicates that coaches should not assume that taller or heavier players are disadvantaged in trunk endurance and should instead focus on consistent development of core stability and fatigue resistance across all types of body.

No significant differences in the plank and Biering-Sørensen tests between high- and low-achieving male players were observed after controlling for height and weight. In contrast significant differences were evident in female players, with higher-ranked girls outperforming lower-ranked peers even after adjusting for body size. This finding is consistent with previous research on female tennis players aged 10–14 years, where higher-ranked athletes demonstrated superior performance in core stability and strength assessments (3). Similarly, Correia (9) reported that higher-rank players who trained more frequently outperformed their lower-rank peers in comparable trunk endurance and stability, reinforcing the link between training exposure, ranking, and physical fitness.

The sex-specific nature of these results warrants attention. An explanation could be that male players, regardless of ranking, may already exhibit relatively homogeneous levels of trunk endurance due to higher habitual participation in strength-orientated training or natural differences in lean body mass and muscle morphology during adolescence (19, 23). In contrast, female players may display greater variability in neuromuscular development and training emphasis, making trunk muscle endurance a more sensitive discriminator of competitive level in this group. It is also possible that differences in training exposure or conditioning emphasis may contribute to these findings as such factors were not directly assessed in the present study.

These findings suggest that trunk conditioning may be relevant, particularly in female players, where endurance performance in plank and Biering-Sørensen tests appears to be linked with competitive success. Considering that trunk endurance contributes not only to performance but also to injury prevention, especially in sports characterised by repetitive rotational loading, such as tennis (7, 15), integrating structured and progressive trunk endurance training into early development programs could have both performance-enhancing and protective benefits. Future longitudinal studies should examine whether improvements in trunk muscle endurance directly translate into better ranking or reduced injury incidence, and whether sex-specific approaches are warranted.

From a practical perspective, the normative values presented in Table 2 may help coaches interpret individual trunk endurance performance relative to players of the same age and sex. Scores below the lower percentiles may indicate a need for additional trunk endurance and stabilization training, while scores around the median may reflect age-appropriate performance. Higher percentile values may indicate well-developed trunk endurance and support progression toward more demanding or tennis-specific conditioning content. Table 2 may serve as a practical reference for screening, monitoring development over time and individualizing training programs in young tennis players.

This study benefits from several methodological strengths such as: relatively large sample size of 214 young tennis players enhances the statistical power and generalizability of the findings, particularly within an athletic population; the stratification of participants based on their national ranking, distinguishing between high- and low-performing players, facilitated a more nuanced analysis of the relationship between playing level and core strength; the utilization of the plank and Biering-Sørensen tests, both well-established and standardized measures of core and trunk muscle endurance, contributes to the enhanced reliability and validity of the obtained results. However, this study also presents several limitations. The cross-sectional design precludes the establishment of causal relationships and the tracking of changes in core strength over time, highlighting the potential benefits of future longitudinal studies. Furthermore, while the study discusses the possible influence of puberty on the results, the absence of direct puberty evaluations or hormonal data weakens the conclusions related to this factor. Trunk endurance tests were performed in a single trial only, which may limit the assessment of within-session reliability and should be considered when interpreting individual performance variability. The limited assessment of participants’ training history beyond playing level restricts the ability to fully explain individual variations in core strength. Finally, the recruitment of participants exclusively from the Croatian Tennis Association introduces a potential selection bias, which can limit the generalizability of the findings to a broader population of young tennis players.

The key findings of the present study indicate that body height and weight were not associated with trunk endurance performance in young tennis players. After adjustment for body size, high-achieving female players demonstrated superior trunk extensor endurance, whereas no significant differences were observed between high- and low-achieving male players. Age and sex-specific normative values for these tests may assist coaches and researchers in evaluating, monitoring, and developing core strength and endurance in youth tennis populations, with potential implications for improving performance and injury prevention.

## Data Availability

The raw data supporting the conclusions of this article will be made available by the authors, without undue reservation.
